# The kinase polypharmacology landscape of clinical PARP inhibitors

**DOI:** 10.1038/s41598-020-59074-4

**Published:** 2020-02-17

**Authors:** Albert A. Antolin, Malaka Ameratunga, Udai Banerji, Paul A. Clarke, Paul Workman, Bissan Al-Lazikani

**Affiliations:** 1 Department of Data Science, The Institute of Cancer Research, London, SM2 5NG UK; 2 Drug Development Unit, The Institute of Cancer Research, London, SM2 5NG UK; 3 Cancer Research UK Cancer Therapeutics Unit, The Institute of Cancer Research, London, SM2 5NG UK

**Keywords:** Cheminformatics, Target identification, Targeted therapies

## Abstract

Polypharmacology plays an important role in defining response and adverse effects of drugs. For some mechanisms, experimentally mapping polypharmacology is commonplace, although this is typically done within the same protein class. Four PARP inhibitors have been approved by the FDA as cancer therapeutics, yet a precise mechanistic rationale to guide clinicians on which to choose for a particular patient is lacking. The four drugs have largely similar PARP family inhibition profiles, but several differences at the molecular and clinical level have been reported that remain poorly understood. Here, we report the first comprehensive characterization of the off-target kinase landscape of four FDA-approved PARP drugs. We demonstrate that all four PARP inhibitors have a unique polypharmacological profile across the kinome. Niraparib and rucaparib inhibit DYRK1s, CDK16 and PIM3 at clinically achievable, submicromolar concentrations. These kinases represent the most potently inhibited off-targets of PARP inhibitors identified to date and should be investigated further to clarify their potential implications for efficacy and safety in the clinic. Moreover, broad kinome profiling is recommended for the development of PARP inhibitors as PARP-kinase polypharmacology could potentially be exploited to modulate efficacy and side-effect profiles.

## Introduction

It is now widely accepted that drugs often bind several proteins beyond their intended target (polypharmacology), which has implications for both therapeutic efficacy and adverse-effects. Accordingly, there is an increasing interest in medicinal chemistry to rationally design multi-target compounds^1–6^. In addition, understanding of polypharmacology can lead to the exploitation of drugs in novel indications, such as the recent approval of crizotinib in ROS1-driven non small cell lung cancer^3,7,8^. In this context, experimental and computational methods are increasingly being used to uncover previously unknown off-targets of drugs^3,9–12^.

**Figure 1:**
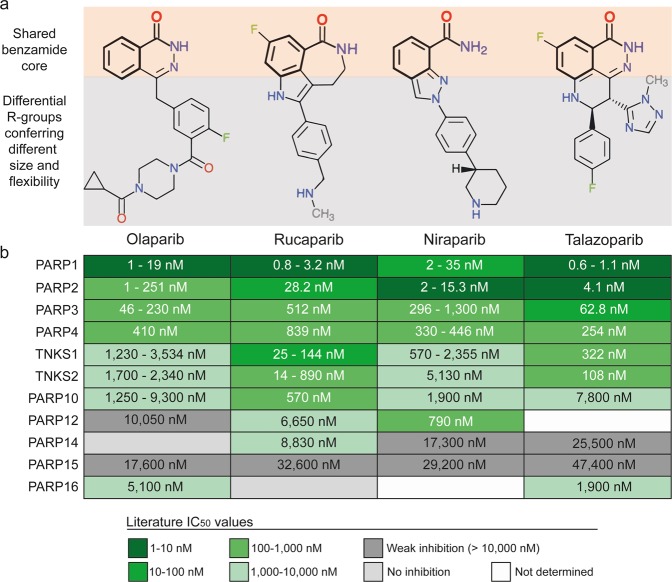
Chemical structures and known PARP activities of FDA-approved PARP inhibitors. (**a)** Chemical structures of the four FDA-approved PARP drugs. The benzamide core pharmacophore shared by all clinical PARP inhibitors is highlighted in bold with orange shading. The rest of the chemical structure that is not shared between the inhibitors and confers them with different size and flexibility has grey shading. (**b)** Known target profile of clinical PARP inhibitors across members of the PARP enzyme family. IC_50_ values are obtained from the literature and the ChEMBL database (www.ebi.ac.uk/chembl/) and ranges are given where there is more than one published value^29,35^.

The demonstration that BRCA1 and BRCA2 mutant human cancer cell lines and tumour xenografts are exquisitely sensitive to small-molecule inhibitors of poly (ADP-ribose) polymerase (PARP) was critical for the clinical development and approval of PARP inhibitors as single agents and provided the first clinical exemplification of synthetic lethality in oncology^13,14^. All FDA-approved PARP inhibitors bind to the nicotinamide binding pocket of PARPs through a shared benzamide pharmacophore that is essential for PARP binding, but the individual agents differ in size and flexibility (Fig. 1a)^15^. In 2014, olaparib was the first PARP inhibitor to be approved by the FDA for advanced BRCA-mutated ovarian cancer, followed by rucaparib which was licensed for the same indication in 2016 (https://www.fda.gov/drugs/resources-information-approved-drugs/hematologyoncology-cancer-approvals-safety-notifications)^16,17^. Niraparib was then approved in 2017 as maintenance treatment for recurrent fallopian tube, ovarian and primary peritoneal cancers (Table 1)^18^. In 2018, olaparib and rucaparib also gained approval as maintenance treatment in the same types of cancer while olaparib was additionally licensed for BRCA-mutated HER2-negative breast cancer (Table 1)^16–18^. Most recently, talazoparib was approved for BRCA-mutated HER2-negative breast cancer^19^ (Table 1). Further PARP inhibitors, including veliparib, are under clinical development^13,20,21^. No strong rationale currently exists for selecting one PARP drug over the others in terms of clinical effectiveness and toxicity and prescription is largely based on the approved indication for each drug as well as the reimbursement policy of the relevant healthcare provider^22,23^. Deeper understanding of the activity and liabilities of individual PARP inhibitors is therefore important to aid clinical decisions and benefit cancer patients as well as to guide the design of future PARP inhibitors.

**Table 1:** Evolution of the indications for FDA-approved PARP inhibitors.

PARP inhibitor	Year of approval	Indication and expanded indication
Olaparib	2014	Treatment of patients with deleterious or suspected deleterious **germline BRCA mutated** (gBRCAm) **advanced ovarian cancer** who have been treated with three or more prior lines of chemotherapy.
	2017	Maintenance treatment of adult patients with recurrent **epithelial ovarian, fallopian tube, or primary peritoneal cancer**, who are in a complete or partial response to platinum-based chemotherapy.
	2018	Treatment of patients with deleterious or suspected deleterious **germline BRCA-mutated** (gBRCAm), HER2-negative metastatic **breast cancer** who have been treated with chemotherapy either in the neoadjuvant, adjuvant, or metastatic setting.
Rucaparib	2016	Treatment of patients with deleterious **BRCA mutation** (germline and/or somatic) associated **epithelial ovarian, fallopian tube or primary peritoneal cancer** who have been treated with two or more chemotherapies.
	2018	Maintenance treatment of recurrent **ovarian, fallopian tube, or primary peritoneal cancer** who are in a complete or partial response to platinum-based chemotherapy.
Niraparib	2017	Maintenance treatment of adult patients with recurrent **epithelial ovarian, fallopian tube, or primary peritoneal cancer** who are in complete or partial response to platinum-based chemotherapy.
Talazoparib	2018	Treatment of patients with deleterious or suspected deleterious **germline BRCA-mutated** (gBRCAm), HER2-negative locally advanced or metastatic **breast cancer**.

Data were extracted from the FDA Hematology/Oncology (Cancer) Approvals & Safety Notifications (accession date 29^th^ May 2018)^65^.

Several differences between individual PARP inhibitors have already been reported at the cellular and clinical levels. When used at micromolar concentrations, differences in DNA strand break repair, phosphorylation of several proteins, cell cycle arrest, and anti-proliferative activities have been described between olaparib, rucaparib and veliparib in cancer cell lines^24,25^. Moreover, the different capacity of PARP inhibitors to trap PARP at the DNA damage site is widely accepted as important for the action of PARP inhibitors. Yet, the exact molecular mechanism of this is not completely understood^26^. Differences in cancer cell sensitivity and synthetic lethality also emerge from recent large-scale profiling experiments, in some instances leading to the prediction of distinct predictive genomic biomarkers (Supplementary Table 1)^27,28^. Overall, it seems important to investigate further the differences between PARP inhibitors and to explore the potential impact on clinical use.

The selectivity and polypharmacology of PARP inhibitors within the PARP-family was recently characterised *in vitro* using an enzymatic inhibition assay^29^. Of the four approved PARP inhibitors, niraparib was shown to be more selective for PARP1 and PARP2 compared to olaparib, rucaparib and talazoparib which show broader pan-PARP activity (Fig. 1b)^29^. However, this differential intra-family PARP selectivity is insufficient to explain all the differences observed between clinical PARP inhibitors. In 2014, we reported for the first time that the different polypharmacology patterns between PARP inhibitors extended beyond the PARP enzyme family^30^. We demonstrated that rucaparib inhibited at least nine kinases with micromolar affinity whereas veliparib inhibited only two kinases and olaparib did not exhibit activity against any of the 16 kinases tested^30^. From a high-throughput screen for RPS6KB1 kinase inhibitors, we identified a series of carboxamidobenzimidazoles that were confirmed to bind RPS6KB1 by orthogonal methods including X-ray crystallography^31^. The carboxamidobenzimidazoles are known inhibitors of PARP^32^ and the existence of a crystal structure of a carboxamidobenzimidazole bound to RPS6KB1 kinase^31^ prompted our speculation that all PARP inhibitors could have an intrinsic capacity to inhibit kinases. This capacity could result from the ability of their shared benzamide pharmacophore to interact with the highly-conserved kinase hinge region (Fig. 1a)^30,31^. Accordingly, depending on its individual molecular size and decoration, each PARP inhibitor could have a unique off-target kinase profile that may remain as yet unexplored and would be important to characterise^4,30^.

More recently, an unbiased, large scale, mass spectrometry-based chemical proteomics approach uncovered new, low-potency affinities of the PARP inhibitor niraparib^33^. However, the chemical proteomics approach used was not able to reproduce published, stronger off-target kinase interactions^30^. This illustrates the limitations of any single method for identifying drug polypharmacology and indicates the need for a more comprehensive analysis^30^.

Here, we objectively assess pharmacological and clinical differences between the four FDA-approved PARP inhibitors, olaparib, rucaparib, niraparib and talazoparib. We use a combination of computational and experimental methods to comprehensively dissect the kinome-wide off target landscape of these PARP inhibitors. We also perform a meta-analysis of FDA approval and key clinical trial data to map the clinically observed adverse effects and hypothesise potential links to the polypharmacology.

## Results

### In silico target profiling predicts new kinase off-targets of clinical PARP inhibitors

We applied three parallel computational methods to predict off-targets: (1) a consensus of six ligand-based chemoinformatic methods integrated in the Chemotargets CLARITY platform^34^; (2) the Similarity Ensemble Approach (SEA)^7^; and (3) the multinomial Naive Bayesian multi-category scikit-learn method implemented in ChEMBL^35^. The common principle for these methods is that chemically similar molecules should share similar bioactivity profiles against molecular targets; however, the details of the methods, including the computational representation (fingerprints) of compounds and similarity calculations used, are distinct. We employed these three computational methods to predict the kinase off-targets of the four FDA-approved PARP inhibitors, olaparib, rucaparib, niraparib and talazoparib. In addition to recovering most of the known interactions with members of the PARP family, the three *in silico* methods predicted a total of 58 potential interactions between PARP inhibitors and kinases, with only 10 of them being previously known^30^ (Table 2, Supplementary Tables 2–4).

**Table 2:** Comparison of the number of kinases predicted for clinical PARP inhibitors using three *in silico* target profiling methods and those experimentally observed by *in vitro* kinome binding at 10 μM.

Method Class	Method	Number of kinases affected			
		Olaparib	Rucaparib	Niraparib	Talazoparib
Computational	CLARITY^34^	23*	7	7	1
	ChEMBL^35^	0	11	3	1
	SEA^66^	0	4	1	0
Experimental	*In vitro* binding (KinomeSCAN®)^38^	0	37	23	2

*Prediction originating from the similarity to a single kinase inhibitor that is likely a false positive.

CLARITY predicted 23 kinases as potential off-targets of olaparib (Supplementary Table 2). However, neither ChEMBL nor SEA predicted any kinase for this PARP drug (Supplementary Tables 3–4). A close inspection of the CLARITY predictions revealed that they were all generated from the similarity of olaparib to a single kinase inhibitor that was likely to be a false positive due to the absence within its structure of a benzamide moiety, which is known to be important for PARP binding (Supplementary Table 2)^15^.

CLARITY predicted seven kinases as potential off-targets of niraparib while ChEMBL predicted three kinases and the SEA method predicted one kinase (Table 2, Supplementary Tables 2–4). However, while all the methods predicted kinases as potential off-targets for niraparib, no two methods predicted the same kinase. The lack of agreement between the methods indicates that niraparib may have general kinase-binding features rather than specific molecular features associated with defined kinases. Interestingly, the only known kinase off-target of niraparib reported previously in the literature had low affinity (DCK IC_50_ = 67.9 μM)^33^. In addition, some of the kinase inhibitors that are identified as similar to niraparib exhibit PARP-binding features. For example, CHEMBL2035040, a weak AKT inhibitor, shares the key benzamide moiety with niraparib and other PARP inhibitors.

We have previously demonstrated that rucaparib inhibits nine kinases with micromolar potency^30^. Given the high degree of polypharmacology of many kinase inhibitors, we hypothesized that rucaparib could inhibit more kinases than the ones already identified. None of the three computational methods used here predicted all of the nine kinase off-targets that are already known. CLARITY, ChEMBL and SEA correctly predicted four, two and four known kinase off-targets, respectively, for rucaparib. Additionally, CLARITY predicted three new kinases, and ChEMBL predicted nine new kinase off-targets (Table 2, Supplementary Tables 2–4). Overall, twelve new kinase off-targets in total were predicted for rucaparib.

Finally, CLARITY and ChEMBL predicted only one kinase off-target each for talazoparib whilst SEA predicted none (Table 2). This low number of kinase off-target predictions suggests that it is less likely that talazoparib inhibits kinases (Supplementary Tables 2–4).

Overall, the lack of consensus on specific kinase off-targets between the three computational methods (Table 2, Supplementary Tables 2–4) is noteworthy and indicates a need for improvement, especially given the expanding use of these computational approaches^36^. It is particularly puzzling given that all three methods are based on chemical structure similarity and use the same underlying medicinal chemistry databases – highlighting the sensitivity of such predictions to the specific computational representation of compounds and statistics utilised. Based on our experience, we advise that, until these methodologies improve, researchers should apply as many predictive computational methods as possible. However, despite the differences in the detail, an important message is that all three methods did predict a range of kinases as potential off-targets of niraparib and rucaparib, thus increasing the confidence in our hypothesis that additional kinase off-targets are likely to be found for PARP drugs (Table 2).

### Kinome profiling with a binding assay uncovers differential polypharmacology between clinical PARP inhibitors

To follow up the computational analysis, we performed a comprehensive *in vitro* kinome screen. To do this we employed the DiscoveRx (https://www.discoverx.com) KinomeScan *in vitro* binding technology^37,38^ that has been widely used for kinome profiling in drug discovery. At the time of conducting our screen, this kinome panel was the largest commercially available and comprised 468 *in vitro* binding assays corresponding to 392 unique human kinases (76% of the human kinome)^39^ (Supplementary Table 5). Also included were assays comprising mutated, deleted, phosphorylated or autoinhibited forms of proteins (n = 63), secondary or pseudokinase domains (n = 8), non-human isoforms (n = 3) and CDK complexes with different cyclins (n = 2) (Supplementary Table 5). The assays were performed at a single relatively high concentration of 10 μM to identify initially both low and high potency off-targets.

**Figure 2:**
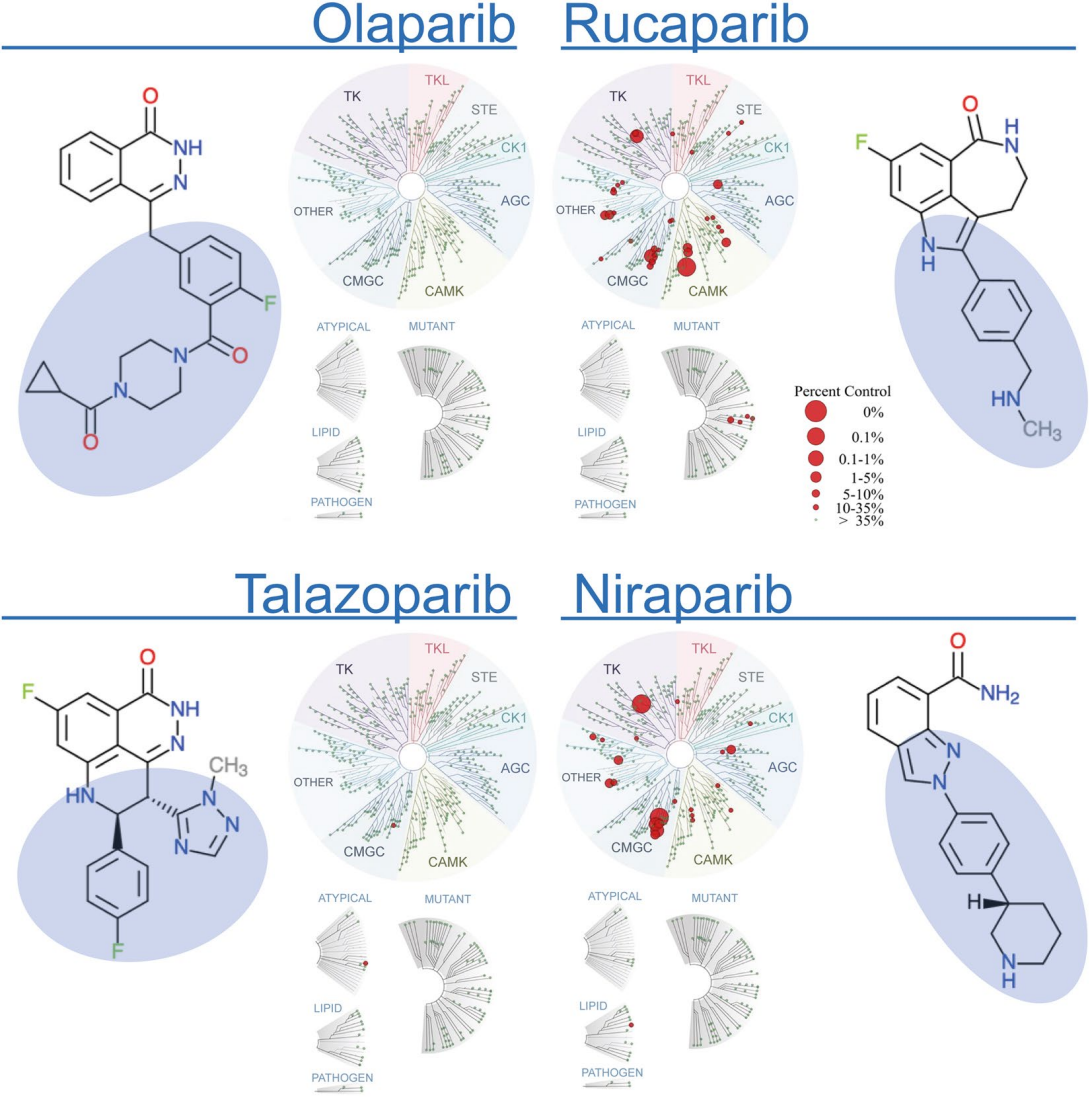
Kinome profiling of the four FDA-approved PARP inhibitors across 392 unique human kinases and 76 mutated, atypical and other forms. This was carried out using the *in vitro* binding platform of DiscoveRx’s KinomeScan®^38^. The assays were perfomed at a single 10 μM concentration. The TREEspot™^64^ representations of the kinome tree, with superimposed *in vitro* binding data for each PARP inhibitor, illustrate how rucaparib and niraparib bind to a significant number of kinases while talazoparib only modestly binds to two kinases and olaparib does not bind to any of the kinases tested. The chemical structures of the PARP drugs are included and their different R-groups highlighted in blue shading to illustrate different side-chains that may influence polypharmacology.

The results of our use of the *in vitro* binding assay for kinome profiling reveal marked differences between the kinase polypharmacology of PARP inhibitors. As illustrated in Fig. 2, rucaparib and niraparib bind to many kinases while talazoparib binds only weakly to two kinases and olaparib does not bind to any of the 392 kinases tested (Supplementary Table 5). The binding technology used involves a competition assay, defining activity as ≥65% of the kinase being competed off an immobilised ligand at 10 μM^38^. Using this measure, rucaparib binds to 37 kinases while niraparib binds to 23 kinases (Table 2, Supplementary Table 5). Moreover, there is only partial overlap between the measured kinase polypharmacology of niraparib and rucaparib, with both drugs binding to 15 shared kinases. Interestingly, the two kinases to which talazoparib showed weak binding are CLK3 and MTOR, neither of which bind the more broadly acting rucaparib and niraparib.

Importantly, as illustrated in Fig. 2, the off-target kinase activities of the four PARP inhibitors do not cluster in one single kinase family but are fairly widely distributed across the kinome. When carried out at the 10 μM concentration used, the *in vitro* binding assay is able to recover most of the previously known off-targets of rucaparib, including the most potent known interactions with PIM1 and DYRK1A. However, the previously described weaker interactions of rucaparib with PRKD2, CDK9, PIM2 and ALK were not reproduced by the binding assay. A further two known off-targets, CDK1 and DCK, were not available in the kinome panel used.

Overall, our results provide empirical evidence that the polypharmacology profile is indeed different for the four different PARP inhibitors studied. Consistent with the prediction by the computational methods, rucaparib and niraparib demonstrate multiple kinase polypharmacology, with fewer or no off-targets for talazoparib and olaparib.

### Orthogonal catalytic inhibition assay confirms DYRK1s, PIM3 and CDK16 as submicromolar off-targets of niraparib and rucaparib

Next, we decided to validate the observed activities in an orthogonal experimental method, namely direct inhibition of kinase catalytic activity using Reaction Biology’s HotSpot platform (http://www.reactionbiology.com)^40^. This platform employs a widely-used and validated radiometric assay which measures inhibition of the incorporation of radiolabelled phosphate into protein substrate^41^. Of the 24 kinases showing the greatest binding by the PARP inhibitors (≥85% binding at 10 μM, Supplementary Table 5) four were not available for follow-up testing with the HotSpot platform (highlighted in Supplementary Table 5). Thus, in total we tested 20 kinases in the radiometric catalytic inhibition assay, initially at a 1 μM concentration of the PARP inhibitors (Table 3). Of these, four were inhibited more than 50% by rucaparib and/or niraparib, namely DYRK1B, CDK16/cyclin Y, PIM3 and DYRK1A (Table 3).

**Table 3:** Validation of the most potent interactions identified in the *in vitro* binding assay at 10 μM (see Methods, Fig. 2, Supplementary Table 5) using an orthogonal assay that directly measures kinase catalytic activity using a widely-validated radiometric assay^40^.

Kinase Group	Gene Name	Kinase complex and aliases	Niraparib	Rucaparib
**CMGC**	**DYRK1B**	**—**	**82%**	**53%**
**CMGC**	**CDK16**	**CDK16/cyclin Y** (PCTAIRE, PCTK1)	n.b.	**82%**
**CAMK**	**PIM3**	**—**	15%	**78%**
**CMGC**	**DYRK1A**	**DYRK1**	**76%**	49%
CMGC	HIPK1	**—**	40%	−16%
CAMK	MYLK4	**—**	19%	36%
Other	AURKB	Aurora B	34%	n.b.
CMGC	HIPK2	**—**	23%	−6%
CAMK	PIM1	**—**	22%	27%*
CMGC	CSNK2A1	CK2a	12%	21%
AGC	LATS2	**—**	17%	n.b.
CMGC	CSNK2A2	CK2a2	−1%	17%
AGC	CIT	STK21	10%	15%
Other	HASPIN	Haspin	10%	−6%
CMGC	CDK4	CDK4/cyclin D3	n.b.	10%
CMGC	HIPK3	**—**	5%	−12%
CAMK	TSSK3	STK22C	n.b.	−1%
PKL	PIK3C3	VPS34	−1%*	n.b.
CAMK	PIM2	**—**	−5%	n.b.
CAMK	STK17A	DRAK1	n.d.	−6%

The table displays the average of duplicate (n = 2) measurements of the percentage of enzyme inhibition relative to DMSO controls sorted by maximum percentage of inhibition. All assays were performed using 1 μM drug concentration and the appropriate Km concentration of ATP. From all the tested kinases, only 4 inhibit the enzyme by >50%. These four most potent interactions, expected to be submicromolar, are displayed at the top of the table and in bold. n.b. not binding. n.d. not determined due to low binding (Supplementary Table 5). * n = 1.

Some of the initial hits identified in the DiscoveRx KinomeScan binding assay – such as TSSK3 – could not be reproduced using the orthogonal radiometric catalytic assay. This is frequently observed when comparing binding and catalytic assays^42^, due to factors such as differences in assay methods and conditions (e.g. protein constructs and drug concentrations used). Interestingly, although the primary hits from the binding assay are widely distributed across the kinome tree (Fig. 2), the 20 selected kinases showing the greatest binding by the PARP inhibitors are from only 5 different kinase groups and the submicromolar kinase off-targets of PARP inhibitors in the radiometric catalytic assay are all from the CMGC and CAMK groups (Table 3). At the submicromolar level tested in the catalytic assay (>50% inhibition at 1 μM), rucaparib inhibited kinases from both groups but niraparib inhibited only two kinases from the CMGC group. DYRK1B was the only kinase inhibited by both rucaparib and niraparib at concentrations below 1 μM (Table 3). These results further emphasize the different kinase polypharmacology behaviour exhibited between these two clinical PARP inhibitors.

**Figure 3:**
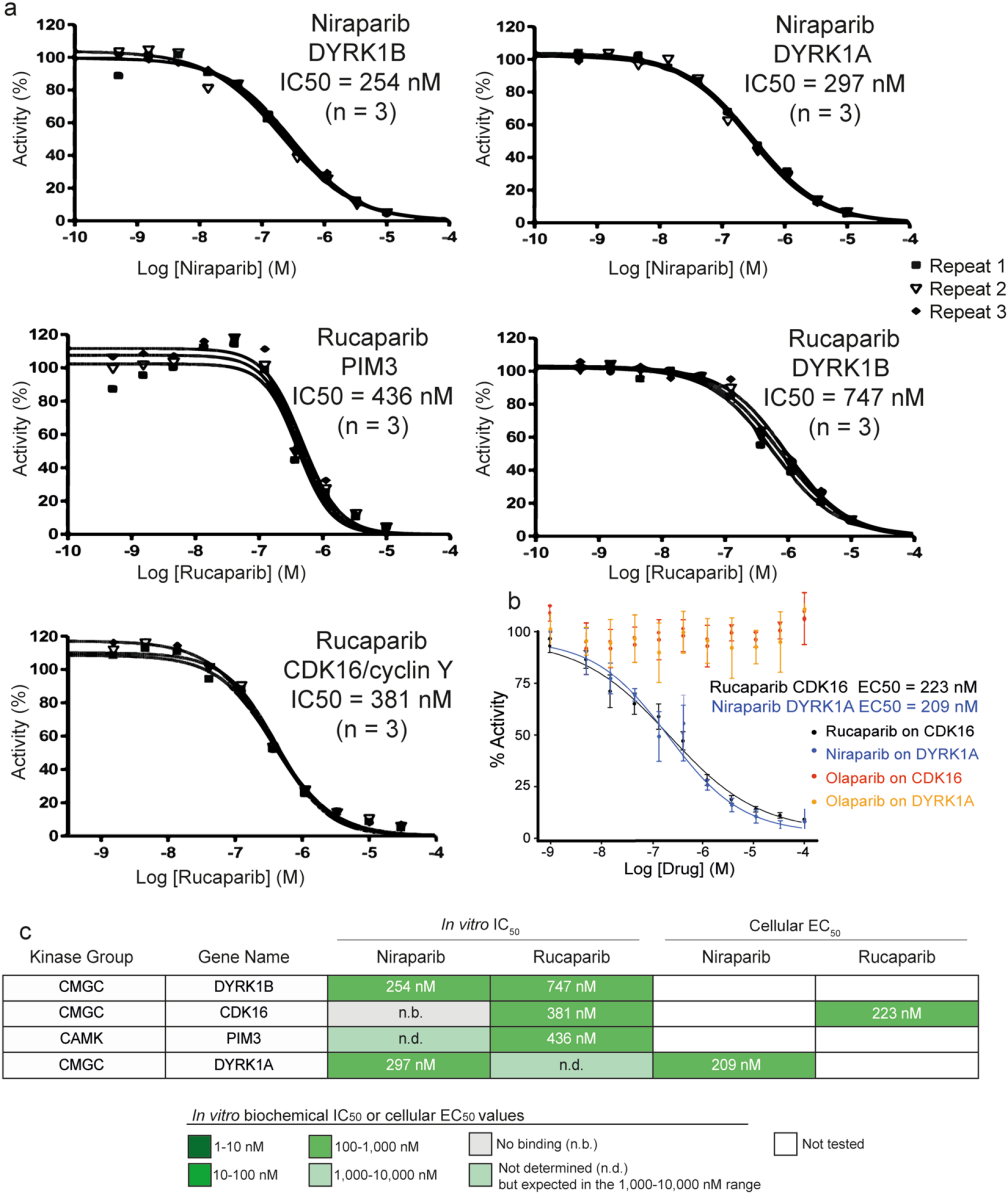
Concentration-response curves for the most potent kinase off-target interactions of clinical PARP inhibitors. (**a)** concentration-response curves and IC_50_ calculation of the most potent interactions in the *in vitro* binding assay (see Methods) of niraparib (top) and rucaparib (bottom) analysed in triplicate using Reaction Biology’s HotSpot radiometric assay that directly measures kinase catalytic activity^40^. (**b)** concentration-response curve and EC_50_ calculation for rucaparib against CDK16 and niraparib against DYRK1A using a target-engagement cellular assay based on NanoBRET technology and an optimized set of cell-permeable kinase tracers (see Methods for details). Olaparib was used as a negative control. The target engagement cellular assays were performed in quadruplicate (two technical repeats in each of two independent experiments). (**c)** table summarising the calculated IC_50_ and EC_50_ values for the kinase off-targets DYRK1B, CDK16, PIM3 and DYRK1A.

Given our identification of potent new submicromolar off-targets that could have clinical implications, we determined the IC_50_ values for kinases showing >50% inhibition at 1 μM using Reaction Biology’s radiometric 10-point concentration-response catalytic inhibition assay (Fig. 3, Supplementary Table 6). Rucaparib inhibits the activity of three kinases (CDK16, PIM3 and DYRK1B) with submicromolar IC_50_ values, the most potent being CDK16 (IC_50_ = 381 nM). In contrast, niraparib inhibits the activity of only two kinases with submicromolar IC_50_ values, the most potent being DYRK1B (IC_50_ = 254 nM). To our knowledge, this is the first report of submicromolar non-PARP family off-targets of PARP inhibitors.

Our docking analysis (see Methods and Supplementary Figs. 1, 2) suggests limited similarity in key contacts between the drugs and their most significant kinase off-targets. All our tested PARP inhibitors, with their varying activities, contain the benzamide moiety which is a key feature for PARP binding. This, together with the lack of predicted common contacts demonstrates that it is unlikely that the benzamide moiety is a major contributor to kinase binding^30^. It is possible that the flatter shape of niraparib and rucaparib might allow a better fit in the kinase binding sites as compared to the bulkier talazoparib and the more flexible olaparib. In turn, this might be responsible for their better docking scores and affinities for kinases. Experimental validation of the predicted binding is required to test these hypotheses.

### Intracellular target engagement confirms submicromolar binding to CDK16 and DYRK1A in transfected HEK293 cells

To test whether the observed biochemical activity translated to cellular activity, we performed intracellular target engagement assays for selected kinases using Reaction Biology’s NanoBRET platform (http://www.reactionbiology.com). From the most potent submicromolar off-targets identified in the biochemical radiometric catalytic assay (Fig. 3), only CDK16 and DYRK1A were available on the NanoBRET platform. We tested rucaparib against CDK16 and niraparib against DYRK1A. We also included olaparib as a negative control. Importantly, the results demonstrate intracellular concentration-responsive binding of both CDK16 by rucaparib and DYRK1A by niraparib in this live cell system (Supplementary Table 7). The EC_50_ values for both interactions are in the 200–230 nM range (niraparib DYRK1A EC_50_ = 209 nM; rucaparib CDK16 EC_50_ = 223 nM). As expected, olaparib does not modulate either of the tested off-target kinases (Fig. 3). Overall, these results further support the off-target inhibition of kinases by some PARP inhibitors at clinically achievable concentrations and in particular reveal kinase engagement in live cells in the submicromolar range.

### Meta-analysis of clinical response and side-effects between PARP inhibitors

Rucaparib and niraparib received FDA-approval in 2016 and 2017 for a recommended dose of 600 mg taken twice daily and 300 mg taken once daily, respectively (Table 1)^17,18^. At these clinical doses, their steady-state Cmax concentrations in plasma range between 2–9 μM for rucaparib^43^ and 3–4 μM for niraparib^44^. These micromolar Cmax concentrations are well above the submicromolar *in vitro* IC_50_ concentrations for their most potent kinase off-targets.

There are no clinical trials comparing PARP inhibitors directly side by side. Registration trials in ovarian cancers for each of the drugs have also been carried out on genetically different population groups. Nonetheless, progression-free survival (PFS) achieved by the intervention arms versus placebo were largely similar for olaparib, rucaparib and niraparib (Hazard ratios (HR) of 0.30, 0.36 and 0.26, respectively). Talazoparib seems to provide the shortest PFS (8.6 months). However, talazoparib was compared to standard of care chemotherapy, achieving an HR of 0.54. In summary, based on existing clinical efficacy data it is difficult to distinguish between the alternative inhibitors (Table 4).

**Table 4:** Clinically observed progression-free survival data for the registration trials of the four FDA approved PARP inhibitors in ovarian and breast cancer.

Drug	Registration trial,	Cohort description & size	Median PFS (Intervention vs Control)	HR	95%CI	P-value
Olaparib	SOLO-1 (NCT01844986) https://www.nejm.org/doi/full/10.1056/NEJMoa1810858	BRCA mutant Olaparib = 260 Placebo = 131	Not reached vs 13.8mo	0.30	0.23–0.41	<0.0001
Rucaparib	ARIEL3 (NCT01968213) https://www.thelancet.com/journals/lancet/article/PIIS0140–6736(17)32440–6/fulltext	Total cohort Rucaparib = 375 Placebo = 189	10.8mo vs 5.4mo	0.36	0.30–0.45	<0.0001
		BRCA mutant Rucaparib = 130 Placebo = 66	16.6mo vs 5.4mo	0.23	0·16–0·34	<0·0001
		Homologous Recombination-deficient Rucaparib = 236 Placebo = 118	13.6mo vs 5.4mo	0·32	0·24–0·42	<0·0001
Niraparib	NOVA (NCT01847274) https://www.nejm.org/doi/full/10.1056/NEJMoa1611310	BRCA mutant Intervention = 138 placebo = 65	21mo vs 5.5mo	0.26	0.17–0.41	<0.0001
Talazoparib	EMBRACA (NCT01945775) https://www.nejm.org/doi/full/10.1056/NEJMoa1802905	BRCA mutant & HER2-negative Talazoparib = 287 SoC = 144	8.6mo vs 5.6mo	0.54	0.41–0.71	<0.0001

To assess adverse reactions, we first used the FDA prescribing information^16–18^ to analyse all 61 reported adverse events and laboratory abnormalities for the four FDA-approved PARP inhibitors (Supplementary Table 8). Given the lack of direct, quantitative, comparative studies, we summarised the findings into qualitative categories to allow comparison (Supplementary Table 9). Next, we abstracted the reported adverse reactions in the four largest clinical trials of olaparib^45^, rucaparib^46^, niraparib^47^ and talazoparib^48^. Despite obvious limitations in comparing different trials, the combination of the abstracted information provides high-level insights into similarities and differences between the different PARP inhibitor side-effects (detailed in Supplementary Tables 8, 9).

**Figure 4:**
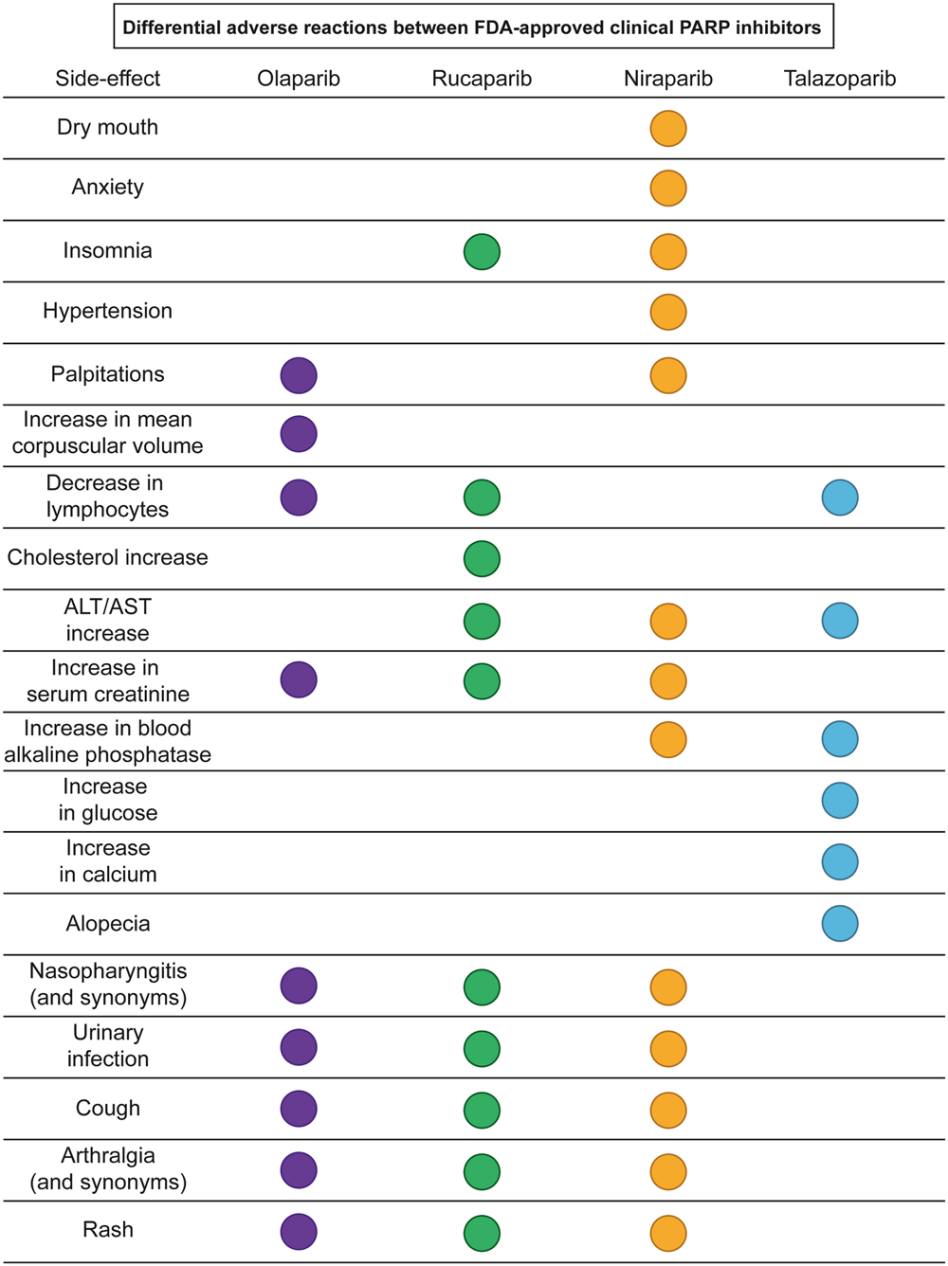
The nineteen differential adverse reactions between FDA-approved clinical PARP inhibitors. Data were extracted from the FDA prescribing information and published results of the largest clinical trials. Side-effect frequencies are not considered due to the differences between the cut-offs used in each trial and FDA prescribing information for each PARP inhibitor (see Methods for details). Each adverse reaction considered common for at least one PARP drug and not identified for at least another PARP inhibitor is represented as a circle. The circles are coloured according to the drugs that present this adverse reaction in their prescribing information or publication of their largest clinical trial.

Of the 61 analysed parameters, 21 are shared between all four approved PARP inhibitors although some are only rarely observed. These include frequently observed side-effects of cancer therapeutics such as nausea, vomiting or diarrhoea. Of the 61 parameters, 40 were reported to be commonly observed side-effects for at least one of the four drugs (Supplementary Table 9). Several of these effects are shared between most PARP drugs, such as the increase in serum creatinine, which is reported during treatment with olaparib, rucaparib and niraparib but not talazoparib (Fig. 4). Other side-effects, such as palpitations, are reported only for two clinical PARP inhibitors. Finally, all four drugs have unique commonly observed side-effects not shared with other PARP inhibitors. For example, rucaparib is the only PARP inhibitor reported to increase cholesterol and talazoparib is the only PARP inhibitor reported to produce alopecia (Fig. 4). All the nineteen distinct side-effects are commonly observed for their respective drugs (as reported in the Prescribing Information). Overall, FDA-approved PARP drugs appear to have distinct side-effect profiles and we hypothesize that their unique polypharmacological profiles could contribute to them.

## Discussion

In this study, we perform a comprehensive computational analysis and experimental characterization of the off-target kinase landscape of the four clinical PARP inhibitors that are currently approved. We demonstrate that each PARP inhibitor has a unique off-target profile across the kinome that should be considered for both the clinical development of PARP inhibitors and the design of new PARP inhibitors with desired polypharmacology. We uncover previously unreported kinase off-targets for the FDA-approved PARP drugs niraparib and rucaparib which we experimentally confirm for the first time to have submicromolar inhibitory activities (Figs. 2 and 3). Niraparib inhibits DYRK1A and DYRK1B whilst rucaparib inhibits CDK16, PIM3 and DYRK1B – all with submicromolar potencies (Fig. 3). We also demonstrate submicromolar intracellular target engagement in live cells for rucaparib binding to CDK16 and niraparib binding to DYRK1A (Fig. 3). We propose that the inhibition by rucaparib and niraparib of DYRK1A/B, CDK16 and PIM3, among other kinases, may have potential clinical relevance and thus warrant further investigation. Moreover, our findings highlight the importance of considering kinase off-targets in the future discovery and development of PARP inhibitors.

Our results illustrate the challenge of comprehensively uncovering drug polypharmacology. We find limitations in all screening assay formats, including chemical proteomics. While computational target prediction methods are increasing in sophistication, we show that they learn general patterns rather than specific details. For example, although all computational methods used here predicted kinases as potential off-targets of PARP inhibitors, the methods showed little overlap in terms of either the precise computational predictions or the results of the experimental measurements (Supplementary Tables 2–6). It is important to note that currently available bioactivity data in public databases are strongly biased towards commonly studied targets, including many kinases. This bias may well contribute to the strong computational prediction of kinase polypharmacology that we observe in the current study. Increasing the target coverage of the public databases will improve the training of computational models. Moreover, current public pharmacological databases focus on the medicinal chemistry literature and data published in journals outside that domain are often missing. For example, the pharmacological data for the previously known kinase off-targets of rucaparib are not available in public databases such as ChEMBL^35^ as they were published in a specialized cancer journal rather than a medicinal chemistry journal^30^. This is an important limitation for the training of computational models. In the present work, we observe that none of the computational methods used is able to recover all the known off-targets of rucaparib because they could not use the above-mentioned profiling data for model building. It is therefore essential that we increase the coverage of public databases to include pharmacological data published outside the medicinal chemistry literature, in order to improve computational methods. Meanwhile, users of such computational methods are advised to apply as many approaches as possible in order to seek a wide range of predictions, and in addition are advised to rely only on those that are experimentally validated.

The availability of experimental *in vitro* biochemical target profiling panels at relatively low cost through contract research organisations (CROs) is strongly enabling – especially democratising off-target identification and validation for smaller enterprises and academic groups. However, technological differences between platforms, including protein expression constructs and systems, purification procedures and assay conditions, affect the results found^42^. In the present study, most of the already known off-targets of PARP inhibitors were reproduced by the *in vitro* competitive binding platform that we used for initial experimental profiling. However, in line with previous observations^42^, some of the strongest binding signals observed with this assay at a compound concentration of 10 µM were not reproduced using a complementary, well-established radiometric catalytic inhibition assay. Moreover, although increasing in coverage, current screening panels are not yet fully comprehensive – even across widely-studied target families such as kinases^49^. Finally, the results of a recent chemical proteomics analysis of the selectivity of clinical PARP inhibitors failed to identify the targets that we discovered and experimentally confirmed – although of course the chemical proteomics technology is able to sample the proteome more broadly^33^. There are several factors limiting the use of chemical proteomics in this setting, such as the level of expression of proteins in the cells used and the unknown full effects of the attached tags across the proteome.

Overall, our results illustrate the complementarity between different methods in addressing the challenging task of systematically uncovering the molecular target profile of drugs to further our understanding of polypharmacology and its potential impact for efficacy and safety in the clinic.

The clinical PARP inhibitors exhibit different sensitivity across cancer cell lines when measured in large-scale screens, enabling the prediction of distinct genomic biomarkers of drug sensitivity (Supplementary Table 1)^27^. It is possible that differential effects between cancer cell lines may relate to the different polypharmacology of PARP inhibitors. We demonstrate intracellular target engagement of CDK16 by rucaparib and DYRK1A by niraparib with EC_50_ values in the 200–230 nM range in live transfected HEK293 cells (Fig. 3). Moreover, the unique capacity of rucaparib to inhibit STAT3 phosphorylation in MDA-MD-231 and MDA-MB-468 human breast cancer cell lines has been previously reported at concentrations below 2.5 μM, but this was not observed in response to olaparib^25^. PIM3 is known to phosphorylate STAT3^50^ and we show that rucaparib is the only FDA-approved PARP inhibitor that inhibits PIM3. These results further support the relevance the kinase polypharmacology of PARP inhibitors in a cellular context. Both the observed differential cellular effects and the differential polypharmacology necessitate consideration of more system-wide effects of PARP inhibitors and their kinase off-targets, particularly in organs that are exposed to high drug concentrations, such as the blood and the liver. Indeed both DYRK1A and CDK16 proteins are highly expressed in the bone marrow, immune cells and the liver while CDK16 is very broadly expressed^51^.

We have demonstrated that rucaparib and niraparib bind their intracellular kinase off-targets at submicromolar concentrations in live cells. However, as shown in Fig. 1, all examined PARP inhibitors have low nanomolar potencies against their PARP targets whereas the potencies against kinases are in the 200 nM to low micromolar range. Moreover, drug penetration inside solid tumours is frequently limited^52^. Therefore, these off-target activities are unlikely to compete directly anti-PARP activity at the tumour site. Yet, the micromolar clinical concentrations achieved, especially at sites of higher drug concentrations including the blood and the liver, may have potential clinical implications with respect to efficacy in haematological cancers and with respect to observed adverse-effects.

Analysing the expression of potent kinase off-targets of PARP inhibitors in haematological cancers using canSAR^53^ reveals that DYRK1A and DYRK1B are overexpressed at the mRNA level in several types of leukaemia and lymphoma (Supplementary Figs. 3–6). Interestingly, DYRK1A has been associated with acute lymphoblastic leukaemia (ALL) in children with Down syndrome (DS), because it is located on chromosome 21 and overexpressed as part of the trisomy that characterizes this disease^54^. Given the high micromolar concentration that niraparib can reach in the blood, this PARP inhibitor could be investigated as a potential repurposing opportunity for this rare paediatric leukaemia^54^.

Our meta-analysis of FDA and clinic-reported adverse events shows that each PARP drug has a unique clinical adverse side-effect pattern and we hypothesize that this may potentially relate, at least in part, to the unique kinase off-target profile (Fig. 4, Supplementary Tables 8, 9). Since adverse side-effects could result through a multitude of mechanisms, it is not possible to ascribe each side-effect to specific kinase off-targets. Nevertheless, the unique inhibition of PIM3 by rucaparib and not by olaparib, niraparib or talazoparib suggests the hypothesis that it may potentially contribute to the unique elevations in cholesterol that are observed in patients treated with rucaparib but not the other three PARP inhibitors (Fig. 3). Interestingly, PIM3 has been recently found to be regulated downstream of mTORC1 by miR-33 – encoded by the SREBP loci^55^. SREBP and miR-33 are known regulators of cholesterol homeostasis^56^. Moreover, transgenic mice overexpressing PIM3 in the liver showed an increase of lipid droplet accumulation^57^ while PIM1 is known to stabilize the cholesterol transporter^55^ and there is substantial functional redundancy in the PIM kinase family. Accordingly, the high drug concentrations that the liver is exposed to and the unique inhibition of PIM3 by rucaparib (Fig. 3) are consistent with the hypothesis that PIM3 kinase inhibition may potentially be responsible for this differential side-effect (Fig. 4). However, further experimental and clinical validation is needed to test this hypothesis, for example by comparing biomarkers of PIM3 response in patients treated with different PARP inhibitors.

The distinct kinase off-target and adverse side-effect profiles between PARP inhibitors that we have identified caution against the assumption that PARP inhibitors are clinically equivalent in all disease and treatment scenarios. Moreover, any differences could be magnified when PARP inhibitors are used in combination with other drugs that could synergise differently with the different kinase off-target activities of clinical PARP inhibitors. This might be particularly important in drug combinations with immunotherapy due to the likely higher concentrations of PARP inhibitors in the blood compared to the tumour site. Currently, there are at least 30 clinical trials studying combinations between PARP inhibitors and immunotherapies (Supplementary Table 10) but none is comparing any PARP inhibitors side by side.

The submicromolar kinase activities that we identify for PARP inhibitors here could potentially have an impact on the complex interplay between PARP inhibitors and immunotherapeutics. For example, the niraparib off-target DYRK1A is known to regulate the branching point between Th17 and Treg differentiation^58^. Therefore, DYRK1A may potentially play a role in increasing the Treg cell population that in turn may antagonise the effects in PARP-immunotherapy drug combinations^58,59^. If this is the case, the combination of olaparib or niraparib with immunotherapy drugs may give different results and we recommend that this should be investigated carefully. More generally, it is possible that some of the potent kinase off-targets of PARP inhibitors reported in this study could modulate T cell homeostasis and the potential implications should be validated further to maximize PARP drug combinations with immunotherapy.

## Conclusions

In summary, our comprehensive computational and experimental analysis demonstrates that PARP inhibitors have an inherent capacity to inhibit kinases off-target and illustrates that each of the clinically approved PARP inhibitors investigated in this work has a unique polypharmacological kinase profile. Our findings emphasize the importance of comprehensive kinase profiling, using orthogonal technologies, of all candidate PARP inhibitors and in addition opens up potential new avenues for the rational design of dual PARP-kinase inhibitors with targeted polypharmacology. Of particular note, we identify novel submicromolar off-target kinases for rucaparib and niraparib. In addition, we demonstrate through our analysis of prescribing information and key clinical trials that FDA-approved PARP drugs have distinct clinical side-effect profiles and we recommend that studies be undertaken to determine the potential contribution of off-target kinase effects to those side-effects. Moreover, we propose that studies be undertaken to explore the potential repurposing of niraparib in paediatric acute lymphoblastic leukemia that occurrs in children with Down syndrome. In addition, our study highlights the field’s currently limited understanding of drug polypharmacology and its implications for efficacy and safety in the clinic. This is particularly important when considering drug combinations, including those involving immunotherapy, with limited understanding of the polypharmacological liabilities of the combined drugs. However, through the application of complementary technologies, we can – as we show here – map key polypharmacological profiles and generate testable hypotheses with clinical potential. In this way, we can help facilitate the maximal exploitation of PARP inhibitors and other drugs for patient benefit.

## Methods

### Drug acquisition

All compounds were purchased from Selleckchem.com

### In silico target profiling

Three computational methods based on chemical similarity were used to predict the kinase off-targets of clinical PARP inhibitors. The canonical SMILES used to define the chemical structures of the four clinical PARP inhibitors analysed were obtained from ChEMBL^35^. The first method used was the predefined consensus of six ligand-based chemoinformatic methods available in the Chemotargets CLARITY platform (https://www.chemotargets.com) and a predefined panel including PARPs and kinases was selected for off-target prediction^34^. Secondly, we used the Similarity Ensemble Approach (SEA) method (http://sea.bkslab.org/) set to default parameters^7^. The third method used was the multinomial Naive Bayesian multi-category scikit-learn similarity-based method implemented in ChEMBL that can be accessed from the ChEMBL website (https://www.ebi.ac.uk/chembl/)^35^. The raw data from the predictions we obtained can be accessed in Supplementary Tables 2–4.

### *In vitro* kinome profiling measuring drug binding

The DiscoveRx KinomeScan platform (https://www.discoverx.com), utilizing an *in vitro* active site-directed competition binding assay, was used to quantitatively measure interactions between the four clinical PARP inhibitors and their largest available kinase panel. At the time when the assays were performed (date: 29/11/2016), 468 *in vitro* binding assays were available, corresponding to 392 unique human kinases (76% of the human kinome)^39^ (Supplementary Table 5).

### *In vitro* kinase radiometric assays

Reaction Biology’s HotSpot platform (http://www.reactionbiology.com)^40^, which employs a radiometric assay to measure phosphorylation of substrate, was used to validate the hits from the kinome binding assay. The radiometric assay is designed to directly detect the true product without the use of modified substrates, coupling enzymes, or detection antibodies. Test or control compounds are incubated with kinase, substrate, cofactors, and radioisotope-labelled ATP (33P-ɣ-ATP). The reaction mixtures are then spotted onto filter papers, which bind the radioisotope-labelled catalytic product, for subsequent measurement. Unreacted phosphate is removed via washing the filters^41^.

### Intracellular target engagement kinase assays

Reaction Biology’s NanoBRET platform (http://www.reactionbiology.com)^60,61^ employs a biophysical technique that enables the quantitative determination of kinase inhibitor occupancy by a ligand in intact living cells. This live cell quantitative capability is achieved via BRET with an optimized set of cell-permeable kinase tracers. The specificity of the BRET signal is dictated by the placement of NanoLuc on the chosen kinase target and transfected into HEK293 cells. These assays were performed at Reaction Biology.

HEK293 human embryonic kidney cells were from ATCC. FuGENER HD Transfection Reagent, KinaseNanoLuc® fusion plasmids, Transfection Carrier DNA, NanoBRET™ Tracer and dilution buffer, NanoBRET™ Nano-Glo® Substrate, Extracellular NanoLuc® Inhibitor were from Promega. Olaparib was always used as a negative control and APY-69 and CEP701 were used as positive controls for the CDK16 and DYRK1A assays, respectively.

HEK293 Cells were transiently transfected with KinaseNanoLuc® Fusion Vector DNA by FuGENER HD Transfection Reagent. Test compounds were delivered into 384 well assay plate using an Echo 550 acoustic dispenser (Labcyte Inc, Sunnyvale, CA). Transfected cells were harvested and mixed with NanoBRET™ Tracer Reagent and dispensed into 384 well plates and incubated the plates at 37 °C in 5% CO_2_ cell culture incubator for 1 hour. The NanoBRET™ Nano-Glo® Substrate plus Extracellular NanoLuc® Inhibitor Solution were added into the wells of the assay plate and incubated for 2–3 minutes at room temperature. The donor emission wavelength (460 nm) and acceptor emission wavelength (600 nm) were measured in an EnVision plate reader. The BRET Ratio were calculated using the equation: BRET Ratio = [(Acceptor sample ÷ Donor sample) – (Acceptor no-tracer control ÷ Donor no-tracer control)].

### Docking experiments

Of the four protein kinases inhibited with submicromolar affinities by rucaparib or niraparib (Fig. 3), only DYRK1A and CDK16 had a 3D structure deposited in the PDB^62^. We selected the crystal structures of DYRK1A (PDB ID: 4AZE) and CDK16 (PDB ID: 3MTL) because they were the only ones co-crystallized with a ligand presenting a cyclic benzamide that mirrors the one present in all PARP inhibitors (Supplementary Table 11). The PDB files were prepared using the standard preparation method implemented in GOLD v5.6 (https://www.ccdc.cam.ac.uk/solutions/csd-discovery/components/gold/). The binding site was described by selecting residues at a distance of 6 Å from the co-crystallized ligand. The ligand structures were extracted from the PDB and edited to define the correct atom types. Docking was performed using standard variables for high conformation sampling (30 GA runs) and amide bond and ring system flexibility of the ligand were enabled. In order to facilitate the analysis of the molecular interactions between PARP inhibitors and kinase proteins in the best GOLD scoring poses, we used LigPlot + to generate 2D schematic diagrams of these protein-ligand interactions^63^.

### Adverse side-effect analysis

FDA prescribing information was downloaded from the FDA website (www.accessdata.fda.gov; accessed: 24/10/2018). The raw data describing the side-effects and laboratory abnormalities and their frequencies were extracted from the FDA prescribing information documents (Supplementary Table 9). A total of 64 side-effects and laboratory abnormalities were described for the four FDA-approved PARP drugs (Supplementary Table 9). From the 64 side-effects, ‘decrease in leucocytes’ and ‘leukopenia’ were considered redundant side-effects and therefore were merged. Similarly, ‘nasopharyngitis/URI/sinusitis/rhinitis/influenza’ and ‘(upper) respiratory tract infection’ were also considered analogous and merged. Finally, ‘ALT increase’ and ‘AST increase’ were also merged in a single side-effect. Accordingly, the final number of side-effects and laboratory abnormalities considered was 61 (Supplementary Table 10). The information was subsequently transformed into a binary format (1 = side-effect present; 0 = side-effect absent) (Supplementary Table 10). Uncommon side-effects by the definition of the FDA label were distinguished from common ones in two different columns (Supplementary Table 10). Differential side-effects were then compared to larger published clinical trials of olaparib, rucaparib and niraparib to make sure they were not observed in more recent and larger clinical trials that may not have been included in the labels^45–47^. Several of the differential side-effects initially identified in the FDA labels were indeed found to have been reported in larger clinical trials, such as dyspepsia, headache or myalgia that had been reported for rucaparib in the latest clinical trial despite not being included in the FDA label (Supplementary Table 9, 10)^46^. Figure. 4 summarizes the nineteen side-effects that are different between PARP inhibitors and that were also reported as common for at least one PARP inhibitor.

## Supplementary information

Supplementary Tables.

Supplementary Figures.

## Data Availability

All data generated or analysed during this study are included in this published article (and its Supplementary Information Files).
