# Supplementary material for: The kinase polypharmacology landscape of clinical PARP inhibitors
Source: Sci Rep. 2020 Feb 17;10:2585. doi: 10.1038/s41598-020-59074-4 (PMC7026418; doi:10.1038/s41598-020-59074-4)

Supplementary Information

The kinase polypharmacology landscape of clinical PARP inhibitors

Albert A. Antolin,[1,2] Malaka Ameratunga,[2] Udai Banerji,[2,3] Paul A. Clarke,[3] Paul Workman[3] and Bissan Al-Lazikani[1,3]

[1] The Department of Data Science, The Institute of Cancer Research, London, SM2 5NG, UK
[2] The Drug Development Unit, The Institute of Cancer Research, London, SM2 5NG, UK
[3] The Cancer Research UK Cancer Therapeutics Unit, The Institute of Cancer Research, London, SM2 5NG, UK

Supplementary Figures

Supplementary	Title	Page #
Supplementary Figure 1	Docking poses with the top GOLD S(PLC) score of four clinical PARP inhibitors in DYRK1A kinase.	3
Supplementary Figure 2	Docking poses with the top GOLD S(PLC) score of four clinical PARP inhibitors in CDK16 kinase.	4
Supplementary Figure 3	Analysis of DYRK1A gene expression (RNA) in lymphoid neoplasm diffuse large B-cell lymphoma across cancer stages defined by AJCC using canSAR.	5
Supplementary Figure 4	Analysis of DYRK1A gene expression (RNA) in acute myeloid leukemia across cancer stages defined by FAB using canSAR.	6
Supplementary Figure 5	Analysis of DYRK1B gene expression in thymoma across cancer stages defined by AJCC using canSAR.	7
Supplementary Figure 6	Analysis of DYRK1B gene expression (RNA) in acute myeloid leukemia across cancer stages defined by FAB using canSAR.	8

Supplementary Figure 1. Docking poses with the top GOLD S(PLC) score of four clinical PARP inhibitors in DYRK1A kinase. In the top left panel, all ligands are superimposed and the whole protein structure is displayed using MacPyMOL (PyMOL v1.8.0.6). In subsequent panels, LigPlot+44 was used to generate schematic diagrams of protein-ligand interactions for the co-crystalized ligand and clinical PARP inhibitors. The lower Table summarises the value of the GOLD scoring function for the highest-scoring pose – the one represented – and the IC_{50} determined in this work (Figure 3) or extracted from the literature for each of the ligands. As it can be observed, docking scores using GOLD's S(PLC) scoring function are concordant with kinase binding affinity. However, no hydrogen-bond interaction was formed in the best scoring GOLD pose of the PARP inhibitors olaparib, rucaparib, niraparib and talazoparib with the kinase. In contrast, the co-crystalized inhibitor forms two hydrogen bonds with DYRK1A.

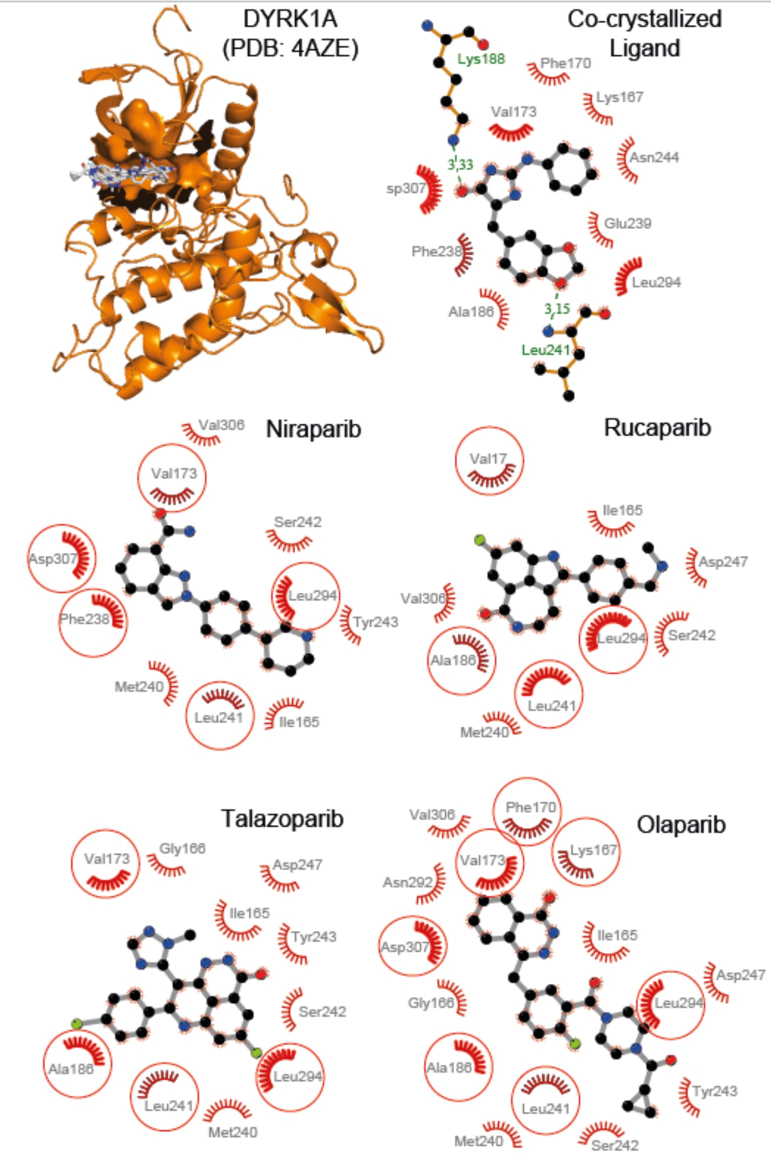

	Co-crystallized Ligand	Niraparib	Rucaparib	Talazoparib	Olaparib
IC50	10-60 nM	297 nM	≈ 1,000 nM	> 10,000 nM	> 10,000 nM
S(PLC)	-60.5	-61.3	-50.8	-44.2	- 42.1

Supplementary Figure 2. Docking poses with the top GOLD S(PLC) score of four clinical PARP inhibitors in CDK16 kinase. In the top left panel, all ligands are superimposed and the whole protein structure is displayed using MacPyMOL (PyMOL v1.8.0.6). In subsequent panels, LigPlot+ was used to generate schematic diagrams of protein-ligand interactions for the co-crystalized ligand and clinical PARP inhibitors. In the bottom, a table summarises the value of the GOLD scoring function for the best pose –that is the one represented– and the IC50 determined in this work (Figure 3) or extracted from the literature for each of the ligands. In this case docking scores are also in line with binding affinity but only rucaparib and talazoparib form one hydrogen bond each while the co-crystalyzed ligand interacted via four hydrogen bonds with CDK16.

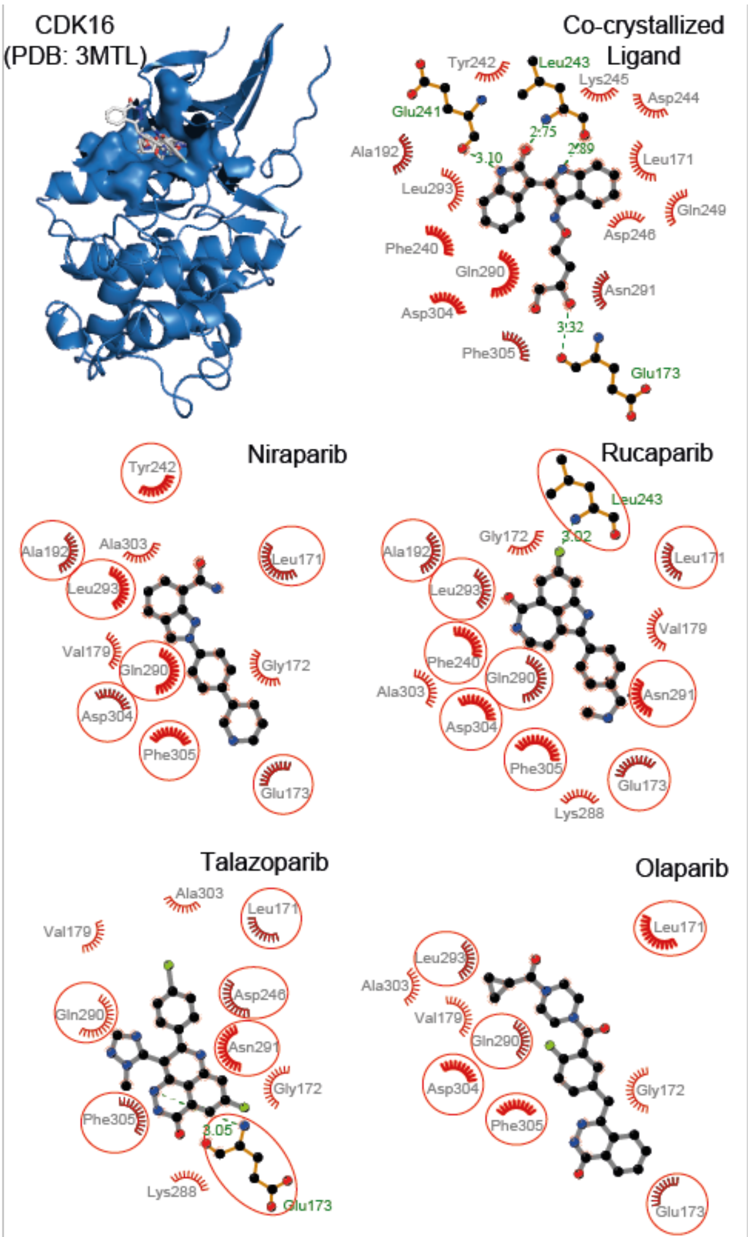

	Co-crystallized Ligand	Niraparib	Rucaparib	Talazoparib	Olaparib
IC50	83 nM	> 10,000 nM	381 nM	> 10,000 nM	> 10,000 nM
S(PLC)	-80.8	-58.0	-60.0	-42.3	- 50.4

Supplementary Figure 3. Analysis of DYRK1A gene expression (RNA) in lymphoid neoplasms diffuse large B-cell lymphoma across cancer stages defined by AJCC (American Joint Committee on Cancer) using canSAR.

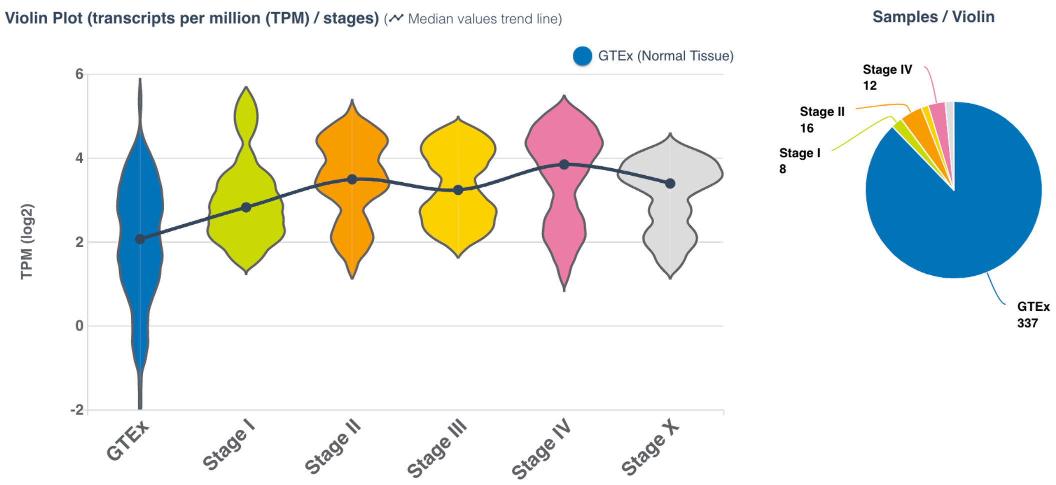

Supplementary Figure 4. Analysis of DYRK1A gene expression (RNA) in acute myeloid leukemia across cancer stages defined by FAB (French-American-British classification system) using canSAR.

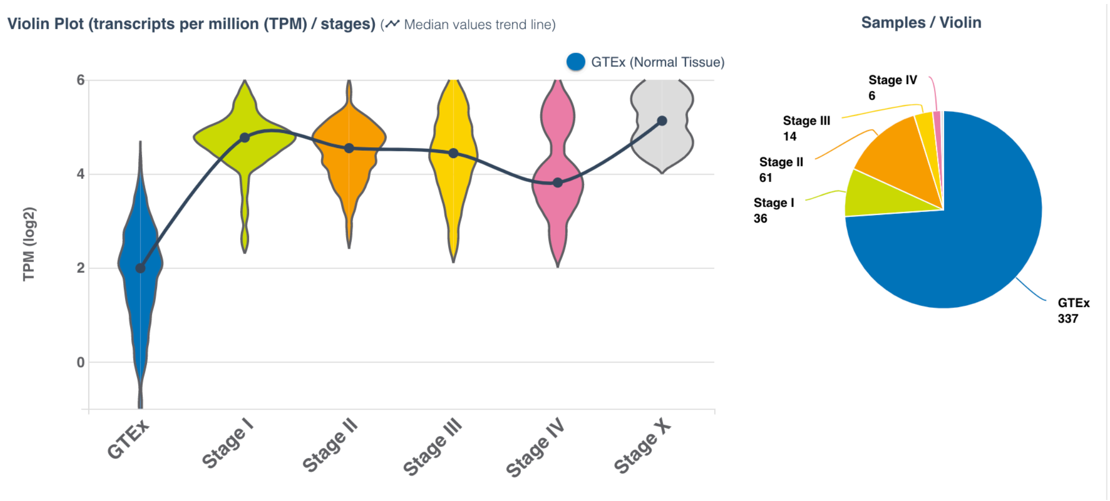

Supplementary Figure 5. Analysis of DYRK1B gene expression (RNA) in thymoma across cancer stages defined by AJCC (American Joint Committee on Cancer) using canSAR.

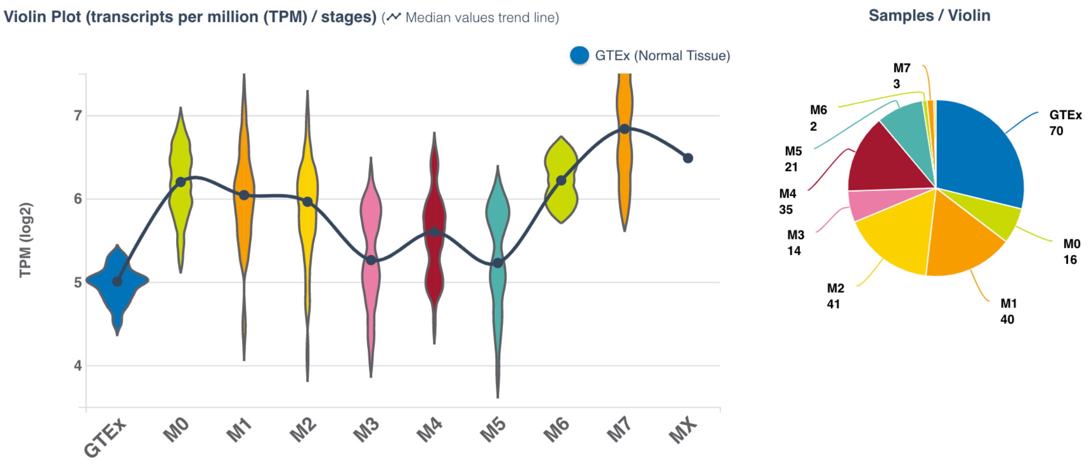

Supplementary Figure 6. Analysis of DYRK1B gene expression (RNA) in acute myeloid leukemia across cancer stages defined by FAB (French-American-British classification system) using canSAR.

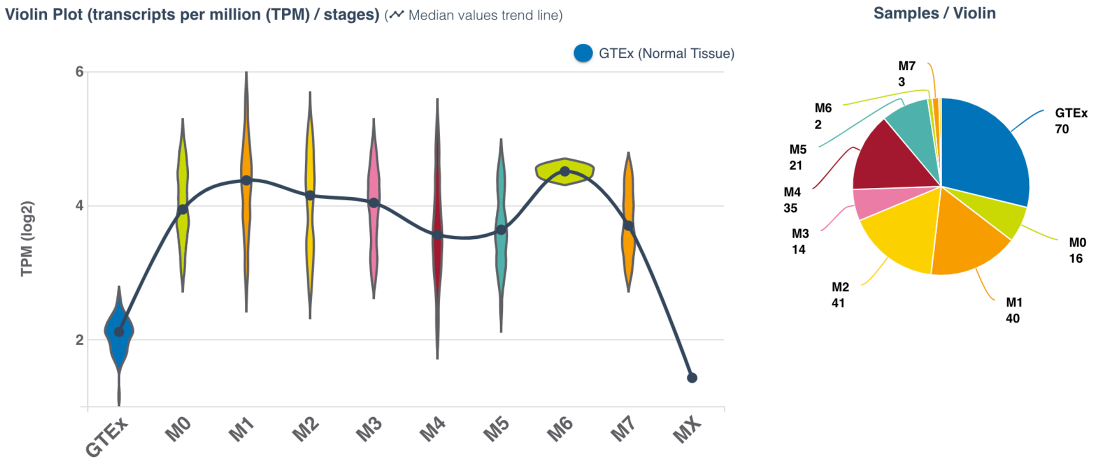

Supplement: Supplementary file 2 — Supplementary Figures. [file 41598_2020_59074_MOESM2_ESM.pdf]
